# Lactate dehydrogenase-to-Albumin Ratio (LAR) predicts mortality in critically ill patients with hypertensive heart failure: A MIMIC-IV database analysis

**DOI:** 10.1016/j.clinsp.2026.100948

**Published:** 2026-04-14

**Authors:** Guang Tu, Zhonglan Cai, Hongke Jiang, Liling Zhang, Huanhuan Hu, Haijian Luo

**Affiliations:** a Department of Cardiology, Lichuan People's Hospital, Fuzhou, China; b Department of Physical Education, Shanghai Maritime University, Shanghai, China; c Department of Cardiovascular Medicine, Anqing Municipal Hospital, Anqing, China; d Department of Cardiology, Central Hospital of Huzhou University, Huzhou, China; e Department of Cardiovascular Medicine, Shanghai Public Health Clinical Center, Fudan University, Shanghai, China; f Department of Internal Medicine and Medical Specialties (DiMI), University of Genoa, Genoa, Italy

**Keywords:** Hypertensive heart failure, Lactate dehydrogenase to albumin ratio, Prognostic marker, Intensive care unit, Mortality Prediction, Critical Illness, Biomarker Ratio, Retrospective Cohort Study

## Abstract

**Background:**

Hypertensive heart disease with heart failure is a severe cardiovascular condition with high mortality rates. The relationship between the Lactate Dehydrogenase to Albumin Ratio (LAR) and mortality in these patients remains unclear.

**Methods:**

The authors analyzed 3019 patients with hypertensive heart failure using data from the MIMIC-IV database. Cox regression models were used to assess the association between LAR and mortality, with adjustments for various covariates. Restricted cubic spline analysis was employed to evaluate the threshold effect of LAR on mortality. Kaplan-Meier survival curves and subgroup analyses further explored the impact of LAR.

**Results:**

LAR was significantly associated with increased mortality across all time points: hospital mortality (HR = 4.86, 95% CI 4.12 to 5.73; p < 0.001), 30-day mortality (HR = 4.16, 95% CI 3.56 to 4.87; p < 0.001), 90-day mortality (HR = 3.56, 95% CI 3.08 to 4.12; p < 0.001), and 365-day mortality (HR = 2.91, 95% CI 2.54 to 3.33; p < 0.001). These associations remained significant after multivariable adjustments. The Cox regression model with restricted cubic spline analysis revealed a threshold effect of LAR on mortality, with turning points at 1.662 for hospital mortality, 2.393 for 30-day mortality, 2.407 for 90-day mortality, and 2.406 for 365-day mortality (all p < 0.001 for non-linear tests). Kaplan-Meier survival curves showed significant differences in survival across LAR quartiles for all mortality outcomes (all p < 0.001).

**Conclusion:**

The LAR is significantly associated with mortality in critically ill patients, with a clear threshold effect. LAR may serve as a potential prognostic marker for identifying high-risk patients.

## Highlights

• LAR is a strong predictor of mortality in ICU patients with hypertensive heart failure.
• Threshold effect identified: LAR >2.4 indicates ultra-high-risk patients.
• Risk increases stepwise across LAR quartiles in Kaplan-Meier survival analysis.
• LAR offers a simple, low-cost prognostic tool from routine lab values.
• Stronger predictive value observed in males and patients with chronic pulmonary disease.

## Introduction

Hypertensive heart disease with heart failure represents a significant public health challenge due to its high prevalence, substantial morbidity, and considerable mortality rates.^1^ This complex condition arises from the interplay of chronic hypertension and subsequent cardiac dysfunction, leading to a progressive decline in cardiac function and frequent hospitalizations.^2^^,^^3^ Despite advancements in medical and surgical interventions, the prognosis for patients with hypertensive heart disease complicated by heart failure remains poor, with high rates of mortality both in the hospital and during long-term follow-up.^4^^,^^5^

The identification of prognostic markers that can reliably predict mortality in these patients is crucial for improving clinical outcomes. Early recognition of high-risk patients allows for more targeted interventions, better resource allocation, and potentially improved survival. Various clinical, laboratory, and imaging parameters have been explored as potential prognostic indicators,^6^^,^^7^ but the search for a robust and reliable marker continues.

Recently, the Lactate dehydrogenase to Albumin Ratio (LAR) has garnered attention as a potential prognostic marker in cardiovascular diseases.^8^^,^^9^ LAR is a composite measure that integrates multiple clinical and laboratory variables to predict the risk of adverse outcomes. Preliminary studies have suggested that LAR might be associated with mortality in different patient populations.^8^, ^9^, ^10^ However, its specific role in hypertensive heart disease with heart failure remains underexplored.

The MIMIC-IV database, a comprehensive and widely-used resource for clinical research, provides a unique opportunity to investigate the relationship between LAR and mortality in a large cohort of patients with hypertensive heart disease complicated by heart failure.^11^ This study aims to fill the gap in the literature by examining the association between LAR and mortality across different time points (hospital mortality, 30-day mortality, 90-day mortality, and 365-day mortality) using robust statistical methods, including multivariable Cox regression models, restricted cubic spline analysis, and Kaplan-Meier survival analysis.

Additionally, subgroup analyses will be performed to explore the differential impact of LAR on mortality across various patient characteristics. This comprehensive approach will provide valuable insights into the potential use of LAR as a prognostic marker in this high-risk patient population.

## Methods

### Data source

This study utilized data from the Medical Information Mart for Intensive Care IV (MIMIC-IV) database, a freely available electronic health record dataset comprising detailed clinical information from patients admitted to the Intensive Care Units (ICUs) of the Beth Israel Deaconess Medical Center.^11^^,^^12^ The authors completed the CITI Data or Specimens Only Research course, obtained approval for database access, and assumed responsibility for data extraction (certification n° 65,828,445).

The MIMIC-IV database includes comprehensive data on demographics, vital signs, laboratory results, diagnoses, and treatments, making it a valuable resource for studying various clinical outcomes.

### Study population

The authors identified patients with a primary diagnosis of hypertensive heart disease complicated by heart failure using relevant International Classification of Diseases, Tenth Revision (ICD-10) codes (I110, I132, 40,291, 40,201, I130, 40,491, 40,493, 40,401, 40,411, 40,211, 40,403, 40,413) from the MIMIC-IV database.^13^

The inclusion criteria were as follows: 1) Age ≥ 18-years, 2) Admission to the ICU, and 3) A diagnosis of hypertensive heart disease complicated by heart failure. Patients with incomplete data on key variables were excluded from the analysis. Due to the absence of left-ventricular ejection fraction or natriuretic peptide data in MIMIC-IV, the authors were unable to classify heart-failure subtypes; thus, the term ‘heart failure’ here refers to clinician-documented decompensation without further phenotyping.

### Variables and definitions

The primary exposure of interest was the LAR, a composite measure derived from clinical and laboratory data. LAR was calculated using the formula: LAR=LactateDehydrogenase(LDH)/Albumin

**Fig. 1:**
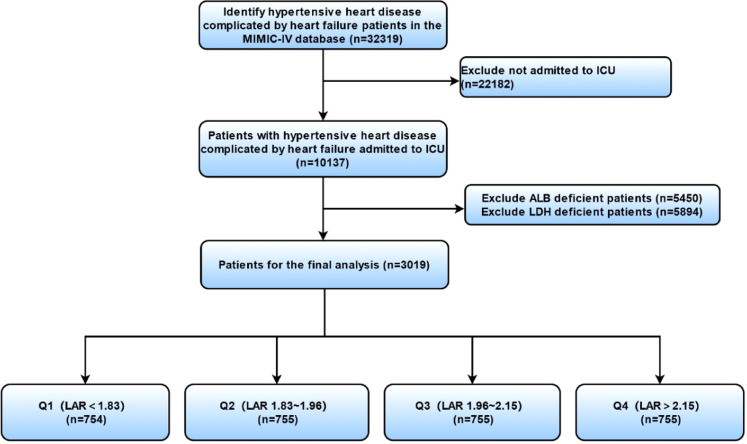
Flowchart of patient inclusion.

The LDH is measured in units of U/L and the albumin in g/dL. Since LAR was not normally distributed, it was log-transformed for analysis. This ratio integrates multiple clinical and laboratory variables to predict the risk of adverse outcomes. The primary outcomes were hospital mortality, 30-day mortality, 90-day mortality, and 365-day mortality. Secondary outcomes included the time to each mortality endpoint (Fig. 1).

Baseline characteristics included demographic data (age, sex, race), comorbid conditions (myocardial infarction, dementia, cerebrovascular disease, chronic pulmonary disease, diabetes, paraplegia, renal disease, metastatic solid tumor), and laboratory measurements (e.g., blood urea nitrogen, creatinine, hemoglobin, platelets, white blood cell count, albumin, anion gap, bicarbonate, glucose, sodium, potassium, INR, PT, APTT, ALT, ALP, AST, total bilirubin, LDH). These variables were extracted from the MIMIC-IV database at the time of ICU admission.

### Statistical analysis

Descriptive statistics summarized the baseline characteristics, with continuous variables presented as mean ± SD or median (IQR) and categorical variables as counts and percentages. Comparisons across LAR quartiles used ANOVA for continuous variables and Chi-Square tests for categorical variables. The association between LAR and mortality was assessed via Cox proportional hazards regression models (unadjusted Model 1, Model 2 adjusted for gender, age, and race, and Model 3 further adjusted for comorbidities). HRs and 95% CIs were reported.

Restricted cubic spline analysis evaluated the threshold effect of LAR on mortality, with Kaplan-Meier curves visualizing survival differences and log-rank tests assessing significance. Subgroup analyses explored the differential impacts of LAR on mortality across patient characteristics, testing interactions in Cox models.

Statistical analyses were conducted using R Statistical Software (Version 4.2.2; The R Project for Statistical Computing) and the Free Statistics Analysis Platform (Version 2.1.1; Beijing, China; http://www.clinicalscientists.cn/freestatistics).^14^

Results with a p-value below 0.05 were considered statistically significant. Data processing and model training were performed on a local server to ensure data security and privacy.

## Results

### Baseline characteristics

A total of 3019 patients with hypertensive heart disease complicated by heart failure were included in the study. The mean age of the study population was 72.5-years (SD = 12.7), with 41.2% being male and 38.2% being white.

Table 1 presents the baseline characteristics of the study population. Significant differences were observed among the quartiles of LAR for several variables, including age, race, heart rate, systolic and diastolic blood pressure, temperature, SpO_2_, hematocrit, hemoglobin, platelets, white blood cell count, albumin, anion gap, bicarbonate, blood urea nitrogen, calcium, chloride, creatinine, glucose, sodium, potassium, INR, PT, APTT, ALT, ALP, AST, total bilirubin, and LDH (all p < 0.05).

**Table 1:** Baseline characteristics of study population.

Variables	Total (n = 3019)	Q1 (n = 754)	Q2 (n = 755)	Q3 (n = 755)	Q4 (n = 755)	p-value
Gender, n (%)						0.186
Male	1243 (41.2)	290 (38.5)	325 (43)	325 (43)	303 (40.1)	
Female	1776 (58.8)	464 (61.5)	430 (57)	430 (57)	452 (59.9)	
Age, years, Mean ± SD	72.5 ± 12.7	72.3 ± 12.9	73.6 ± 13.3	72.8 ± 12.2	71.5 ± 12.3	0.009
Race, n (%)						<0.001
White	1154 (38.2)	258 (34.2)	271 (35.9)	280 (37.1)	345 (45.7)	
Non-White	1865 (61.8)	496 (65.8)	484 (64.1)	475 (62.9)	410 (54.3)	
Heart rate, beats/min, Mean ± SD	71.1 ± 16.3	69.4 ± 15.6	70.5 ± 15.4	71.4 ± 16.2	73.2 ± 17.6	<0.001
Systolic blood pressure, mmHg, Mean ± SD	88.7 ± 18.4	92.7 ± 18.4	90.1 ± 17.7	86.9 ± 17.2	85.0 ± 19.4	<0.001
Diastolic blood pressure, mmHg, Mean ± SD	46.7 ± 12.3	47.9 ± 12.1	47.0 ± 11.6	45.8 ± 11.8	46.0 ± 13.6	0.004
Temperature, °C, Mean ± SD	36.3 ± 0.8	36.4 ± 0.6	36.4 ± 0.5	36.3 ± 0.6	36.2 ± 1.2	<0.001
SpO_2_, %, Mean ± SD	89.5 ± 8.0	90.1 ± 7.5	90.2 ± 6.1	89.8 ± 7.2	88.1 ± 10.5	<0.001
Hematocrit, %, Mean ± SD	29.4 ± 7.1	30.3 ± 6.9	29.9 ± 7.1	29.1 ± 6.9	28.4 ± 7.2	<0.001
Hemoglobin, g/dL, Mean ± SD	9.3 ± 2.3	9.6 ± 2.4	9.4 ± 2.3	9.2 ± 2.3	9.0 ± 2.4	<0.001
Platelets, × 10^9^/L, Mean ± SD	188.0 ± 102.8	195.0 ± 93.7	193.5 ± 93.4	193.5 ± 116.6	170.0 ± 103.6	<0.001
White blood cell count, × 10^9^/L, Median (IQR)	9.5 (7.0, 13.2)	8.3 (6.1, 10.9)	9.1 (6.8, 12.3)	10.1 (7.5, 13.9)	11.6 (8.1, 15.6)	<0.001
Albumin, g/dL, Mean ± SD	3.1 ± 0.6	3.5 ± 0.5	3.2 ± 0.6	3.0 ± 0.6	2.8 ± 0.6	<0.001
Anion gap, mmol/L, Mean ± SD	13.5 ± 4.3	12.8 ± 3.7	13.1 ± 3.7	13.4 ± 4.1	14.9 ± 5.1	<0.001
Bicarbonate, mmol/L, Mean ± SD	20.7 ± 6.1	22.4 ± 5.7	22.1 ± 5.8	20.6 ± 5.7	17.9 ± 6.1	<0.001
Blood urea nitrogen, mg/dL, Median (IQR)	31.0 (19.0, 51.0)	29.0 (18.0, 47.0)	30.0 (19.0, 47.0)	33.0 (20.0, 53.0)	34.0 (21.0, 57.0)	<0.001
Calcium, mmol/L, Mean ± SD	8.3 ± 0.8	8.6 ± 0.7	8.4 ± 0.7	8.2 ± 0.8	8.0 ± 0.8	<0.001
Chloride, mmol/L, Mean ± SD	98.0 ± 7.2	97.8 ± 6.9	98.0 ± 6.9	98.4 ± 7.5	97.9 ± 7.5	0.503
Creatinine, mg/dL, Median (IQR)	1.5 (1.0, 2.5)	1.4 (0.9, 2.5)	1.4 (0.9, 2.3)	1.5 (1.0, 2.6)	1.6 (1.1, 2.6)	<0.001
Glucose, mg/dL, Mean ± SD	125.6 ± 53.7	122.1 ± 52.4	124.6 ± 45.6	124.8 ± 47.9	131.0 ± 66.3	0.010
Sodium, mmol/L, Mean ± SD	135.8 ± 6.0	135.9 ± 5.5	136.1 ± 6.0	135.9 ± 5.9	135.3 ± 6.4	0.056
Potassium, mmol/L, Mean ± SD	4.1 ± 0.6	4.1 ± 0.6	4.1 ± 0.6	4.0 ± 0.6	4.1 ± 0.7	0.090
INR, Mean ± SD	1.6 ± 0.8	1.5 ± 0.7	1.5 ± 0.7	1.6 ± 0.7	1.8 ± 1.1	<0.001
PT, seconds, Mean ± SD	17.5 ± 9.1	16.0 ± 7.8	16.5 ± 7.6	17.5 ± 8.0	19.8 ± 11.7	<0.001
APTT, seconds, Mean ± SD	35.0 ± 16.6	33.6 ± 15.0	33.1 ± 14.3	36.6 ± 18.9	36.7 ± 17.3	<0.001
ALT, U/L, Median (IQR)	22.0 (13.0, 47.0)	16.0 (11.0, 25.0)	18.0 (12.0, 31.0)	24.0 (15.0, 47.0)	54.0 (23.0, 188.8)	<0.001
ALP, U/L, Median (IQR)	90.0 (67.0, 131.0)	87.0 (65.0, 117.0)	89.0 (67.0, 119.0)	93.0 (69.0, 136.0)	95.0 (69.0, 158.0)	< 0.001
AST, U/L, Median (IQR)	32.0 (20.0, 68.0)	20.0 (15.0, 28.0)	25.0 (18.0, 38.0)	37.0 (24.0, 67.0)	105.0 (45.0, 365.0)	< 0.001
Total bilirubin, mg/dL, Median (IQR)	0.6 (0.4, 1.0)	0.5 (0.3, 0.8)	0.5 (0.4, 0.9)	0.6 (0.4, 1.1)	0.8 (0.5, 1.5)	<0.001
LDH, U/L, Median (IQR)	288.0 (215.0, 421.5)	186.0 (160.0, 213.0)	255.0 (223.5, 289.0)	337.0 (288.0, 388.0)	652.0 (502.0, 968.5)	<0.001
Myocardial infarction, n (%)						<0.001
No	1876 (62.1)	535 (71)	512 (67.8)	427 (56.6)	402 (53.2)	
Yes	1143 (37.9)	219 (29)	243 (32.2)	328 (43.4)	353 (46.8)	
Dementia, n (%)						0.548
No	2790 (92.4)	700 (92.8)	696 (92.2)	690 (91.4)	704 (93.2)	
Yes	229 (7.6)	54 (7.2)	59 (7.8)	65 (8.6)	51 (6.8)	
Cerebrovascular disease, n (%)						0.105
No	2548 (84.4)	646 (85.7)	625 (82.8)	625 (82.8)	652 (86.4)	
Yes	471 (15.6)	108 (14.3)	130 (17.2)	130 (17.2)	103 (13.6)	
Chronic pulmonary disease, n (%)						<0.001
No	2043 (67.7)	484 (64.2)	485 (64.2)	527 (69.8)	547 (72.5)	
Yes	976 (32.3)	270 (35.8)	270 (35.8)	228 (30.2)	208 (27.5)	
Diabetes, n (%)						0.124
No	2116 (70.1)	540 (71.6)	541 (71.7)	531 (70.3)	504 (66.8)	
Yes	903 (29.9)	214 (28.4)	214 (28.3)	224 (29.7)	251 (33.2)	
Paraplegia, n (%)						0.015
No	2881 (95.4)	706 (93.6)	717 (95)	730 (96.7)	728 (96.4)	
Yes	138 (4.6)	48 (6.4)	38 (5)	25 (3.3)	27 (3.6)	
Renal disease, n (%)						0.255
No	1369 (45.3)	325 (43.1)	336 (44.5)	345 (45.7)	363 (48.1)	
Yes	1650 (54.7)	429 (56.9)	419 (55.5)	410 (54.3)	392 (51.9)	
Metastatic solid tumor, n (%)						0.021
No	2890 (95.7)	733 (97.2)	728 (96.4)	717 (95)	712 (94.3)	
Yes	129 (4.3)	21 (2.8)	27 (3.6)	38 (5)	43 (5.7)	

Notes: SD stands for Standard Deviation, IQR for Interquartile Range, INR for International Normalized Ratio, PT for Prothrombin Time, APTT for Activated Partial Thromboplastin Time, ALT for Alanine Aminotransferase, ALP for Alkaline Phosphatase, AST for Aspartate Aminotransferase, and LDH for Lactate Dehydrogenase.

### Association between LAR and mortality

In the unadjusted model (Model 1), LAR was significantly associated with increased mortality across all time points: hospital mortality (HR = 4.86, 95% CI 4.12 to 5.73; p < 0.001), 30-day mortality (HR = 4.16, 95% CI 3.56 to 4.87; p < 0.001), 90-day mortality (HR = 3.56, 95% CI 3.08 to 4.12; p < 0.001), and 365-day mortality (HR=2.91, 95% CI 2.54 to 3.33; p < 0.001) (Table 2).

**Table 2:** A multivariate Cox regression model evaluated the association between LAR and mortality in patients with hypertensive heart disease complicated by heart failure.

Variable	No	N, event (%)	Model 1		Model 2		Model 3	
			HR (95% CI)	p-value	HR (95% CI)	p-value	HR (95%CI)	p-value
All-cause mortality within hospital								
LAR	3019	726 (24)	4.86 (4.12∼5.73)	<0.001	5.35 (4.52∼6.33)	<0.001	5.37 (4.53∼6.37)	<0.001
LAR								
Q1	754	89 (11.8)	1 (Ref)		1 (Ref)		1 (Ref)	
Q2	755	118 (15.6)	1.37 (1.04∼1.8)	0.026	1.33 (1.01∼1.75)	0.041	1.32 (1∼1.74)	0.047
Q3	755	188 (24.9)	2.32 (1.8∼2.98)	<0.001	2.33 (1.81∼3)	<0.001	2.36 (1.83∼3.04)	<0.001
Q4	755	331 (43.8)	4.87 (3.85∼6.15)	<0.001	5.07 (4.01∼6.41)	<0.001	5.33 (4.2∼6.77)	<0.001
Trend.test	3019	726 (24)	1.76 (1.64∼1.89)	<0.001	1.8 (1.67∼1.93)	<0.001	1.84 (1.7∼1.98)	<0.001
All-cause mortality within 30 days								
LAR	3019	894 (29.6)	4.16 (3.56∼4.87)	<0.001	4.72 (4.02∼5.55)	<0.001	4.74 (4.02∼5.58)	<0.001
LAR								
Q1	754	128 (17)	1 (Ref)		1 (Ref)		1 (Ref)	
Q2	755	158 (20.9)	1.27 (1.01∼1.61)	0.042	1.24 (0.98∼1.56)	0.076	1.23 (0.97∼1.55)	0.088
Q3	755	242 (32.1)	2.09 (1.69∼2.59)	<0.001	2.11 (1.7∼2.61)	<0.001	2.11 (1.7∼2.62)	<0.001
Q4	755	366 (48.5)	3.8 (3.11∼4.65)	<0.001	4.04 (3.3∼4.95)	<0.001	4.2 (3.42∼5.15)	<0.001
Trend.test	3019	894 (29.6)	1.61 (1.51∼1.71)	<0.001	1.65 (1.55∼1.76)	<0.001	1.68 (1.57∼1.79)	<0.001
All-cause mortality within 90 days								
LAR	3019	1178 (39)	3.56 (3.08∼4.12)	<0.001	4.04 (3.48∼4.68)	<0.001	4.07 (3.5∼4.74)	<0.001
LAR								
Q1	754	191 (25.3)	1 (Ref)		1 (Ref)		1 (Ref)	
Q2	755	239 (31.7)	1.3 (1.08∼1.58)	0.006	1.27 (1.05∼1.53)	0.015	1.26 (1.04∼1.53)	0.017
Q3	755	320 (42.4)	1.91 (1.6∼2.29)	<0.001	1.94 (1.63∼2.33)	<0.001	1.95 (1.62∼2.33)	<0.001
Q4	755	428 (56.7)	3.15 (2.65∼3.73)	<0.001	3.36 (2.83∼3.99)	<0.001	3.47 (2.92∼4.13)	<0.001
Trend.test	3019	1178 (39)	1.48 (1.41∼1.57)	<0.001	1.53 (1.45∼1.61)	<0.001	1.55 (1.46∼1.64)	<0.001
All-cause mortality within 365 days								
LAR	3019	1552 (51.4)	2.91 (2.54∼3.33)	<0.001	3.28 (2.86∼3.77)	<0.001	3.33 (2.89∼3.83)	<0.001
LAR								
Q1	754	300 (39.8)	1 (Ref)		1 (Ref)		1 (Ref)	
Q2	755	337 (44.6)	1.18 (1.01∼1.38)	0.033	1.15 (0.99∼1.35)	0.076	1.16 (0.99∼1.35)	0.069
Q3	755	415 (55)	1.63 (1.41∼1.9)	<0.001	1.67 (1.44∼1.94)	<0.001	1.68 (1.44∼1.95)	<0.001
Q4	755	500 (66.2)	2.47 (2.14∼2.85)	<0.001	2.64 (2.29∼3.05)	<0.001	2.75 (2.37∼3.18)	<0.001
Trend.test	3019	1552 (51.4)	1.37 (1.3∼1.43)	<0.001	1.4 (1.34∼1.47)	<0.001	1.42 (1.36∼1.49)	<0.001

Notes: HR stands for Hazard Ratio, CI for Confidence Interval, Ref for Reference category. Model 1 is the unadjusted model. Model 2 is adjusted for gender, age, and race. Model 3 is adjusted for gender, age, race, myocardial infarction, dementia, cerebrovascular disease, chronic pulmonary disease, diabetes, paraplegia, renal disease, and metastatic solid tumor.

After adjusting for gender, age, and race (Model 2), the associations remained significant: hospital mortality (HR = 5.35, 95% CI 4.52 to 6.33; p < 0.001), 30-day mortality (HR = 4.72, 95% CI 4.02 to 5.55; p < 0.001), 90-day mortality (HR = 4.04, 95% CI 3.48 to 4.68; p < 0.001), and 365-day mortality (HR = 3.28, 95% CI 2.86 to 3.77; p < 0.001) (Table 2).

Further adjustment for myocardial infarction, dementia, cerebrovascular disease, chronic pulmonary disease, diabetes, paraplegia, renal disease, and metastatic solid tumor (Model 3) yielded similar results: hospital mortality (HR = 5.37, 95% CI 4.53 to 6.37; p < 0.001), 30-day mortality (HR = 4.74, 95% CI 4.02 to 5.58; p < 0.001), 90-day mortality (HR = 4.07, 95% CI 3.50 to 4.74; p < 0.001), and 365-day mortality (HR = 3.33, 95% CI 2.89 to 3.83; p < 0.001) (Table 2). Trend tests for LAR quartiles showed a significant increasing trend in mortality across all time points (all p < 0.001) (Table 2).

### Threshold effect of LAR on mortality

**Fig. 2:**
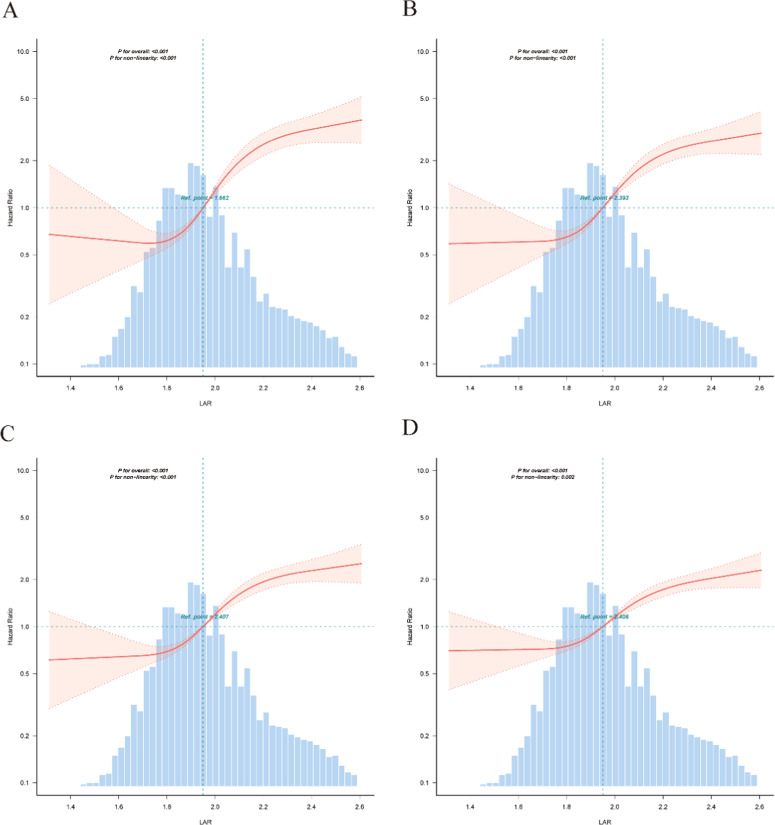
**RCS curve for the LAR.** (A) Restricted cubic spline curve for hospital mortality in patients with hypertensive heart disease complicated by heart failure, (B) Restricted cubic spline curve for 30-day mortality in patients with hypertensive heart disease complicated by heart failure, (C) Restricted cubic spline curve for 90-day mortality in patients with hypertensive heart disease complicated by heart failure, (D) Restricted cubic spline curve for 365-day mortality in patients with hypertensive heart disease complicated by heart failure.

The Cox regression model with restricted cubic spline analysis revealed a threshold effect of LAR on mortality. The turning points for LAR were identified as follows: 1.662 for hospital mortality, 2.393 for 30-day mortality, 2.407 for 90-day mortality, and 2.406 for 365-day mortality. Non-linear tests for all models were significant (all p < 0.001), indicating a threshold effect of LAR on mortality (Table 3 and Fig. 2).

**Table 3:** Cox regression model was used to analyze the threshold effect of LAR on mortality.

Item	HR (95% CI)	p-value
All-cause mortality within hospital		
Turning point	1.662 (1.585, 1.739)	
LAR < 1.622	3033.437 (0.045, 203,632,150.653)	0.157
LAR >= 1.622	13.801 (9.753, 19.531)	<0.001
Non-linear Test*2		<0.001
All-cause mortality within 30 days		
Turning point	2.393 (2.337, 2.45)	
LAR < 2.393	14.564 (9.844, 21.547)	<0.001
LAR >= 2.393	1.125 (0.017, 72.937)	0.956
Non-linear Test*2		<0.001
All-cause mortality within 90 days		
Turning point	2.407 (2.341, 2.474)	
LAR < 2.407	9.002 (6.478, 12.508)	<0.001
LAR >= 2.407	0.082 (0.001, 7.713)	0.281
Non-linear Test*2		<0.001
All-cause mortality within 365 days		
Turning point	2.406 (2.34, 2.471)	
LAR < 2.406	6.069 (4.564, 8.069)	<0.001
LAR >= 2.406	0.226 (0.004, 14.156)	0.481
Non-linear Test*2		0.002

Notes: HR represents Hazard Ratio, and CI represents Confidence Interval. The turning point indicates the threshold value of LAR, where the relationship between LAR and hospital mortality changes. The non-linear test assesses the significance of the threshold effect.

### Kaplan-Meier survival analysis

**Fig. 3:**
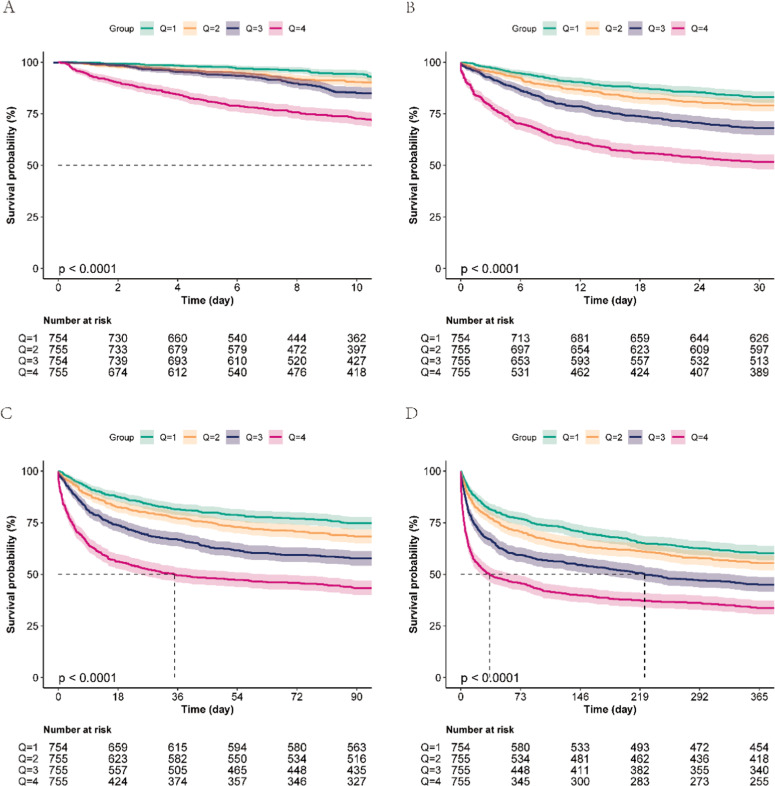
**Kaplan-Meier survival analysis curves for mortality.** LAR (quartile): Q1 (< 1.83), Q2 (1.83∼1.96), Q3 (1.96∼2.15), Q4 (> 2.15). (A) Kaplan-Meier curve for hospital mortality in patients with hypertensive heart disease complicated by heart failure, (B) Kaplan-Meier curve for 30-day mortality in patients with hypertensive heart disease complicated by heart failure, (C) Kaplan-Meier curve for 90-day mortality in patients with hypertensive heart disease complicated by heart failure, (D) Kaplan-Meier curve for 365-day mortality in patients with hypertensive heart disease complicated by heart failure.

Kaplan-Meier survival curves demonstrated significant differences in survival across LAR quartiles for hospital mortality, 30-day mortality, 90-day mortality, and 365-day mortality (all p < 0.001). The survival curves showed a clear gradient of increasing mortality with higher LAR quartiles (Fig. 3).

### Forest plot for subgroup analysis

**Fig. 4:**
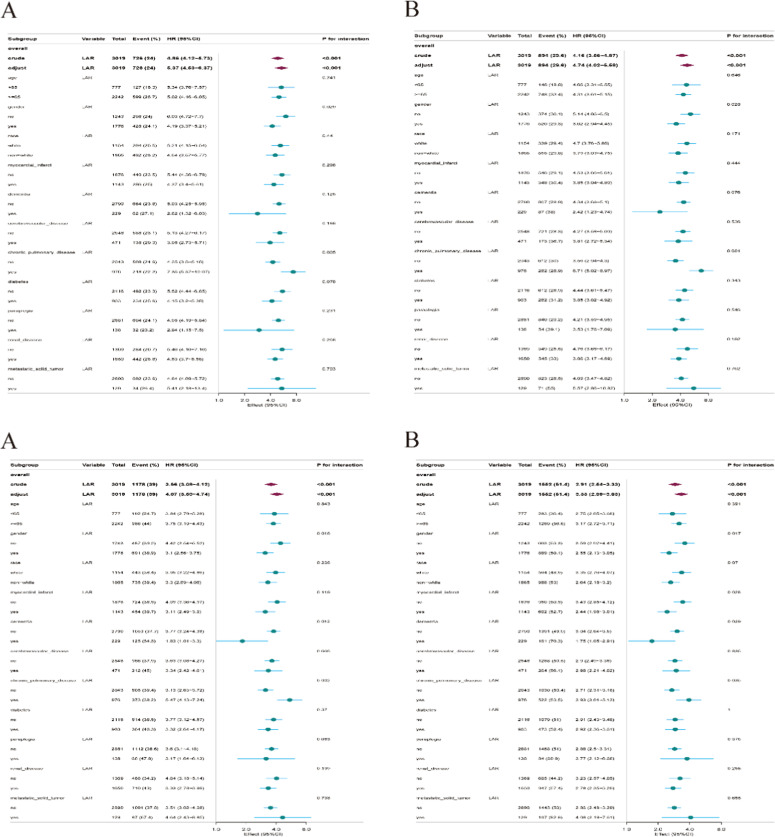
**Forest plot for the subgroup analysis of the relationship between mortality and LAR.** (A) Forest plot for the subgroup analysis of the relationship between hospital mortality and LAR, (B) Forest plot for the subgroup analysis of the relationship between 30-day mortality and LAR, (C) Forest plot for the subgroup analysis of the relationship between 90-day mortality and LAR, (D) Forest plot for the subgroup analysis of the relationship between 365-day mortality and LAR.

Forest plots for subgroup analysis indicated significant interactions for gender (p = 0.029 for hospital mortality, p = 0.028 for 30-day mortality, p = 0.016 for 90-day mortality, and p = 0.017 for 365-day mortality) and chronic pulmonary disease (p = 0.005 for hospital mortality, p = 0.001 for 30-day mortality, p = 0.002 for 90-day mortality, and p = 0.035 for 365-day mortality) (Fig. 4 and Additional Tables S6‒S9).

## Discussion

The present study provides robust evidence that the LAR is a significant predictor of mortality in patients with hypertensive heart disease complicated by heart failure. Specifically, LAR was found to be significantly associated with mortality across multiple time points, including hospital mortality, 30-day mortality, 90-day mortality, and 365-day mortality. This association remained significant even after adjusting for multiple confounding factors, highlighting the potential utility of LAR as a prognostic marker in this high-risk patient population.

Furthermore, this analysis revealed a threshold effect of LAR on mortality, indicating that patients with LAR values above specific thresholds may be at higher risk for adverse outcomes. These findings offer clinicians a new tool for early identification of high-risk patients, potentially improving patient outcomes.

Previous studies exploring prognostic markers in patients with hypertensive heart disease complicated by heart failure have often been limited by small sample sizes, lack of comprehensive covariate adjustment, and a focus on single-time-point mortality outcomes.^15^, ^16^, ^17^ These limitations may have affected the reliability and generalizability of their findings. In contrast, the present study leveraged the extensive and detailed data available in the MIMIC-IV database, allowing for a more comprehensive analysis.

The authors employed robust statistical methods, including multivariable Cox regression models and restricted cubic spline analysis, to account for potential confounders and explore non-linear relationships. This approach provided a more nuanced understanding of the association between LAR and mortality compared to prior studies that may have overlooked the complexity of this relationship.

The predictive power of LAR may be attributed to its integration of multiple key clinical and laboratory variables, particularly Lactate Dehydrogenase (LDH) and Albumin (ALB).^18^^,^^19^ LDH is an enzyme widely present in human tissues, and its levels significantly increase in response to tissue damage or hypoxia, serving as an important marker of cellular injury and inflammatory response.^20^

In patients with heart failure, elevated LDH levels may reflect myocardial ischemia, inflammatory response, or systemic inflammation.^20^, ^21^, ^22^ ALB, on the other hand, is a major plasma protein whose levels are often associated with the patient's nutritional status and liver function.^23^ Low ALB levels may indicate malnutrition, chronic inflammation, or liver dysfunction, all of which are associated with adverse outcomes in heart failure patients.^24^^,^^25^

From a biological perspective, LAR, by combining LDH and ALB, provides a composite indicator that can reflect the patient's overall inflammatory state and nutritional status.^26^ Higher LAR values may indicate more severe inflammatory responses and/or malnutrition, which could lead to higher mortality risks.^27^ Additionally, the threshold effect of LAR further suggests that when LAR exceeds specific thresholds, patient mortality risk significantly increases.

This finding implies that LAR may influence the prognosis of heart failure patients by reflecting an imbalance in inflammation and nutritional status.^28^ This mechanism is likely closely related to the pathophysiological processes of heart failure, as both inflammation and malnutrition have been shown to be associated with the progression and adverse outcomes of heart failure.^29^

### Strengths of the study

A major strength of this study is the use of the MIMIC-IV database, which provides a large, diverse, and well-characterized cohort of patients. This allowed us to conduct analyses with substantial statistical power and adjust for a wide range of potential confounders, thereby enhancing the robustness of the present findings. The Strengthening the Reporting of Observational Studies in Epidemiology (STROBE) statement is followed.

Additionally, the comprehensive approach, including multivariable Cox regression models, restricted cubic spline analysis, and subgroup analyses, provided a detailed and nuanced understanding of the relationship between LAR and mortality.

These methods not only revealed the threshold effect of LAR but also explored its differential impact across various patient subgroups, offering valuable insights for clinical risk stratification and targeted interventions. By identifying high-risk patients early, clinicians can implement more aggressive management strategies, potentially improving outcomes.

### Limitations and efforts

Despite these strengths, the present study has several limitations. First, the retrospective nature of the study and reliance on electronic health record data may introduce biases related to data quality and completeness. To mitigate this, the authors conducted rigorous data cleaning and excluded patients with incomplete data on key variables.

Moreover, while the authors adjusted for multiple confounders, residual confounding may still exist due to unmeasured variables. Importantly, the present analysis did not directly compare the prognostic performance of LAR with established biomarkers such as NT-proBNP, hs-cTn, or ST2; this comparison is a key area for future validation and is required before LAR can be recommended for routine clinical use. To address this, the authors used a comprehensive set of covariates based on prior literature and clinical relevance. On the other hand, the generalizability of these findings may be limited to the specific patient population included in the MIMIC-IV database. Future studies should validate the authors’ findings in other cohorts and settings.

Finally, the causal relationship between LAR and mortality cannot be established from the authors’ observational study design. Due to the absence of left-ventricular ejection fraction or natriuretic peptide data in MIMIC-IV, the authors were unable to classify heart-failure subtypes; thus, the term ‘heart failure’ here refers to clinician-documented decompensation without further phenotyping. Prospective studies and clinical trials are needed to further explore the potential causal mechanisms and therapeutic implications of these findings.

## Conclusions

The present study demonstrates that the LAR is a significant predictor of mortality in hypertensive heart disease with heart failure. LAR, by integrating LDH and ALB, reflects the patient's systemic inflammatory state and nutritional status, potentially playing a significant role in the pathophysiological processes of heart failure.

Future research should further investigate the underlying mechanisms of LAR and validate these findings in larger, more diverse cohorts to provide stronger support for clinical practice. By identifying high-risk patients early, clinicians can implement more aggressive management strategies, potentially improving patient outcomes.

## Consent for publication

Not applicable.

## Disclosures

All the authors, including Guang Tu, Zhonglan Cai, Hongke Jiang, Liling Zhang, Huanhuan Hu, and Haijian Luo have nothing to disclose.

## Compliance with ethics guidelines

The research adhered to the principles outlined in the 1964 Helsinki Declaration and subsequent revisions. MIMIC-IV serves as a de-identified public dataset. Approval for this endeavor was granted by the review boards of both the Massachusetts Institute of Technology (MIT) and Beth Israel Deaconess Medical Center (BIDMC), with an exemption from obtaining informed consent. MIMIC-IV public database of clinical investigations is exempt from the Institutional Review Board (IRB) requirements.

## Data availability statement

The data used in this study are available from the Massachusetts Institute of Technology (MIT) and Beth Israel Deaconess Medical Center (BIDMC) upon request.

## Abbreviations

LAR, Lactate Dehydrogenase to Albumin Ratio; LDH, Lactate Dehydrogenase; MIMIC-IV, Medical Information Mart for Intensive Care IV; HR, Hazard Ratio; CI, Confidence Interval; ICD-10, International Classification of Diseases, Tenth Revision; ICU, Intensive Care Unit; SpO_2_, Peripheral Capillary Oxygen Saturation; INR, International Normalized Ratio; PT, Prothrombin Time; APTT, Activated Partial Thromboplastin Time; ALT, Alanine Aminotransferase; ALP, Alkaline Phosphatase; AST, Aspartate Aminotransferase; LDH, Lactate Dehydrogenase; Q1-Q4, Quartiles 1 to 4; SD, Standard Deviation; IQR, Interquartile Range.

## Authors’ contributions

Conception and design: Haijian Luo, Guang Tu. Administrative support: Haijian Luo, Guang Tu. Provision of study materials or patients: Zhonglan Cai, Hongke Jiang. Collection and assembly of data: Guang Tu, Zhonglan Cai, Hongke Jiang, Liling Zhang, Huanhuan Hu. Data analysis and interpretation: Zhonglan Cai, Hongke Jiang, Liling Zhang, Huanhuan Hu. Manuscript writing: Haijian Luo, Guang Tu. Manuscript review: Haijian Luo. Final approval of manuscript: All authors.

## Funding

Clinical Research Project of Shanghai Public Health Clinical Center, Fudan University, China (KY-GW-2023–05).

## Conflicts of interest

The authors declare that they have no known competing financial interests or personal relationships that could have appeared to influence the work reported in this paper.

## Appendix Supplementary materials

Image, application 1

Image, application 2
