# Supplementary material for: Lactate dehydrogenase-to-Albumin Ratio (LAR) predicts mortality in critically ill patients with hypertensive heart failure: A MIMIC-IV database analysis
Source: Clinics (Sao Paulo). 2026 Apr 14;81:100948. doi: 10.1016/j.clinsp.2026.100948 (PMC13092872; doi:10.1016/j.clinsp.2026.100948)
Supplement: Supplementary file 1 [file mmc1.docx]

CLINICS-D-25-01077_Supplementary Material

**Additional Table S1** The meaning of ICD-9 and ICD-10 codes for disease.

| **Variables** | **Diagnostic code (icdCode)** | **Meaning of diagnostic code** |
| --- | --- | --- |
| Hypertensive heart disease with heart failure | I110 | Hypertensive heart disease with heart failure |
|  | I132 | Hypertensive heart and chronic kidney disease with heart failure and with stage 5 chronic kidney disease, or end stage renal disease |
|  | 40291 | Unspecified hypertensive heart disease with heart failure |
|  | 40201 | Malignant hypertensive heart disease with heart failure |
|  | I130 | Hypertensive heart and chronic kidney disease with heart failure and stage 1 through stage 4 chronic kidney disease, or unspecified chronic kidney disease |
|  | 40491 | Hypertensive heart and chronic kidney disease, unspecified, with heart failure and with chronic kidney disease stage I through stage IV, or unspecified |
|  | 40493 | Hypertensive heart and chronic kidney disease, unspecified, with heart failure and chronic kidney disease stage V or end stage renal disease |
|  | 40401 | Hypertensive heart and chronic kidney disease, malignant, with heart failure and with chronic kidney disease stage I through stage IV, or unspecified |
|  | 40411 | Hypertensive heart and chronic kidney disease, benign, with heart failure and with chronic kidney disease stage I through stage IV, or unspecified |
|  | 40211 | Benign hypertensive heart disease with heart failure |
|  | 40403 | Hypertensive heart and chronic kidney disease, malignant, with heart failure and with chronic kidney disease stage V or end stage renal disease |
|  | 40413 | Hypertensive heart and chronic kidney disease, benign, with heart failure and chronic kidney disease stage V or end stage renal disease |

**Additional Table S2** A univariate Cox regression model evaluated the association between LAR and hospital mortality in patients with hypertensive heart disease complicated by heart failure.

| **Item** | **HR (95% CI)** | **p-value** |
| --- | --- | --- |
| Gender |  |  |
| Male vs. Female | 0.994 (0.8573, 1.1524) | 0.936 |
| Age (years) | 1.02 (1.01, 1.03) | <0.001 |
| Race |  |  |
| White vs. Non-White | 0.91 (0.78, 1.05) | 0.196 |
| Heart rate (beats/min) | 1.0029 (0.9983, 1.0075) | 0.220 |
| Systolic blood pressure (mmHg) | 0.97 (0.97,0.97) | <0.001 |
| Diastolic blood pressure (mmHg) | 0.96 (0.95, 0.97) | <0.001 |
| Temperature (°C) | 0.84 (0.8, 0.87) | <0.001 |
| SpO_2_ (%) | 0.97 (0.96, 0.97) | <0.001 |
| Hematocrit (%) | 0.97 (0.96, 0.98) | <0.001 |
| Hemoglobin (g/dL) | 0.89 (0.86, 0.92) | <0.001 |
| Platelets (×10⁹/L) | 0.9983 (0.9975, 0.9991) | <0.001 |
| White blood cell count (×10⁹/L) | 1.01 (1.01, 1.02) | <0.001 |
| Albumin (g/dL) | 0.45 (0.4, 0.5) | <0.001 |
| Anion gap (mmol/L) | 1.11 (1.1, 1.13) | <0.001 |
| Bicarbonate (mmol/L) | 0.93 (0.91, 0.94) | <0.001 |
| Blood urea nitrogen (mg/dL) | 1.0095 (1.0072, 1.0118) | <0.001 |
| Calcium (mmol/L) | 0.77 (0.7, 0.84) | <0.001 |
| Chloride (mmol/L) | 0.99 (0.98, 1) | 0.009 |
| Creatinine (mg/dL) | 1.08 (1.05, 1.12) | <0.001 |
| Glucose (mg/dL) | 1.003 (1.0017, 1.0042) | <0.001 |
| Sodium (mmol/L) | 0.9971 (0.985, 1.0093) | 0.637 |
| Potassium (mmol/L) | 1.17 (1.04, 1.31) | 0.008 |
| INR | 1.22 (1.17, 1.28) | <0.001 |
| PT (seconds) | 1.02 (1.02, 1.03) | <0.001 |
| APTT (seconds) | 1.0088 (1.0055, 1.0121) | <0.001 |
| ALT (U/L) | 1.0003 (1.0002, 1.0004) | <0.001 |
| ALP (U/L) | 1.0015 (1.0012, 1.0019) | <0.001 |
| AST (U/L) | 1.0001 (1.0001, 1.0002) | <0.001 |
| Total bilirubin (mg/dL) | 1.09 (1.07, 1.11) | <0.001 |
| LDH (U/L) | 1.0002 (1.0002, 1.0002) | <0.001 |
| Myocardial infarction |  |  |
| Yes vs. No | 1.1 (0.95, 1.28) | 0.190 |
| Dementia |  |  |
| Yes vs. No | 1.18 (0.91, 1.53) | 0.219 |
| Cerebrovascular disease |  |  |
| Yes vs. No | 1.33 (1.11, 1.6) | 0.002 |
| Chronic pulmonary disease |  |  |
| Yes vs. No | 0.89 (0.76, .04) | 0.138 |
| Diabetes |  |  |
| Yes vs. No | 1.12 (0.95, 1.3) | 0.170 |
| Paraplegia |  |  |
| Yes vs. No | 1.0017 (0.7028, 1.4277) | 0.992 |
| Renal disease |  |  |
| Yes vs. No | 1.34 (1.16, 1.56) | <0.001 |
| Metastatic solid tumor |  |  |
| Yes vs. No | 1.24 (0.88, 1.74) | 0.227 |
| LAR | 4.86 (4.12, 5.73) | <0.001 |

**Additional Table S3** A univariate Cox regression model evaluated the association between LAR and 30-day mortality in patients with hypertensive heart disease complicated by heart failure

| **Item** | **HR (95% CI)** | **p-value** |
| --- | --- | --- |
| Gender |  |  |
| Male vs. Female | 0.96 (0.84, 1.1) | 0.562 |
| Age (years) | 1.03 (1.02, 1.03) | <0.001 |
| Race |  |  |
| White vs Non-White | 1.01 (0.88, 1.16) | 0.881 |
| Heart rate (beats/min) | 1.0037 (0.9996, 1.0079) | 0.080 |
| Systolic blood pressure (mmHg) | 0.97 (0.97, 0.98) | <0.001 |
| Diastolic blood pressure (mmHg) | 0.96 (0.96, 0.97) | <0.001 |
| Temperature (°C) | 0.84 (0.81, 0.88) | <0.001 |
| SpO₂ (%) | 0.97 (0.96, 0.97) | <0.001 |
| Hematocrit (%) | 0.97 (0.96, 0.98) | <0.001 |
| Hemoglobin (g/dL) | 0.9 (0.88,0.93) | <0.001 |
| Platelets (×10⁹/L) | 0.9985 (0.9977, 0.9992) | <0.001 |
| White blood cell count (×10⁹/L) | 1.01 (1.01, 1.01) | <0.001 |
| Albumin (g/dL) | 0.49 (0.44, 0.54) | <0.001 |
| Anion gap (mmol/L) | 1.1 (1.09, 1.12) | <0.001 |
| Bicarbonate (mmol/L) | 0.94 (0.93, 0.95) | <0.001 |
| Blood urea nitrogen (mg/dL) | 1.0096 (1.0075, 1.0116) | <0.001 |
| Calcium (mmol/L) | 0.83 (0.77, 0.9) | <0.001 |
| Chloride (mmol/L) | 0.99 (0.98, 1) | 0.022 |
| Creatinine (mg/dL) | 1.08 (1.05, 1.12) | <0.001 |
| Glucose (mg/dL) | 1.0023 (1.0011, 1.0035) | <0.001 |
| Sodium (mmol/L) | 0.9976 (0.9867, 1.0086) | 0.671 |
| Potassium (mmol/L) | 1.25 (1.13, 1.38) | <0.001 |
| INR | 1.22 (1.17, 1.27) | <0.001 |
| PT (seconds) | 1.02 (1.02, 1.02) | <0.001 |
| APTT (seconds) | 1.0087 (1.0058, 1.0117) | <0.001 |
| ALT (U/L) | 1.0002 (1.0001, 1.0003) | <0.001 |
| ALP (U/L) | 1.0016 (1.0013, 1.0019) | <0.001 |
| AST (U/L) | 1.0001 (1.0001, 1.0002) | <0.001 |
| Total bilirubin (mg/dL) | 1.08 (1.06, 1.1) | <0.001 |
| LDH (U/L) | 1.0002 (1.0001, 1.0002) | <0.001 |
| Myocardial infarction |  |  |
| Yes vs. No | 1.09 (0.95, 1.24) | 0.230 |
| Dementia |  |  |
| Yes vs. No | 1.36 (1.09, 1.7) | 0.006 |
| Cerebrovascular disease |  |  |
| Yes vs. No | 1.37 (1.16, 1.62) | <0.001 |
| Chronic pulmonary disease |  |  |
| Yes vs. No | 0.95 (0.83, 1.1) | 0.494 |
| Diabetes |  |  |
| Yes vs. No | 1.08 (0.94, 1.24) | 0.282 |
| Paraplegia |  |  |
| Yes vs. No | 1.41 (1.07, 1.85) | 0.015 |
| Renal disease |  |  |
| Yes vs. No | 1.35 (1.18, 1.54) | <0.001 |
| Metastatic solid tumor |  |  |
| Yes vs. No | 2.19 (1.71, 2.79) | <0.001 |
| LAR | 4.16 (3.56, 4.87) | <0.001 |

**Additional Table S4** A univariate Cox regression model evaluated the association between LAR and 90-day mortality in patients with hypertensive heart disease complicated by heart failure.

| **Item** | **HR (95% CI)** | **p-value** |
| --- | --- | --- |
| Gender |  |  |
| Male vs. Female | 0.98 (0.87, 1.1) | 0.748 |
| Age (years) | 1.03 (1.02, 1.03) | <0.001 |
| Race |  |  |
| White vs. Non-White | 1.03 (0.91, 1.16) | 0.657 |
| Heart rate (beats/min) | 1.0047 (1.0011, 1.0084) | 0.011 |
| Systolic blood pressure (mmHg) | 0.98 (0.97, 0.98) | <0.001 |
| Diastolic blood pressure (mmHg) | 0.97 (0.96,0.97) | <0.001 |
| Temperature (°C) | 0.85 (0.82, 0.88) | <0.001 |
| SpO_2_ (%) | 0.97 (0.97, 0.98) | <0.001 |
| Hematocrit (%) | 0.97 (0.97, 0.98) | <0.001 |
| Hemoglobin (g/dL) | 0.91 (0.89, 0.94) | <0.001 |
| Platelets (×10⁹/L) | 0.9986 (0.998, 0.9992) | <0.001 |
| White blood cell count (×10⁹/L) | 1.0095 (1.0057, 1.0132) | <0.001 |
| Albumin (g/dL) | 0.52 (0.47, 0.57) | <0.001 |
| Anion gap (mmol/L) | 1.09 (1.07, 1.1) | <0.001 |
| Bicarbonate (mmol/L) | 0.95 (0.94, 0.96) | <0.001 |
| Blood urea nitrogen (mg/dL) | 1.0088 (1.007, 1.0106) | <0.001 |
| Calcium (mmol/L) | 0.86 (0.8, 0.93) | <0.001 |
| Chloride (mmol/L) | 0.9911 (0.9833, 0.9991) | 0.028 |
| Creatinine (mg/dL) | 1.06 (1.03, 1.09) | <0.001 |
| Glucose (mg/dL) | 1.0017 (1.0006, 1.0028) | 0.002 |
| Sodium (mmol/L) | 0.9952 (0.9857, 1.0048) | 0.329 |
| Potassium (mmol/L) | 1.19 (1.09, 1.3) | <0.001 |
| INR | 1.22 (1.17, 1.27) | <0.001 |
| PT (seconds) | 1.02 (1.02, 1.02) | <0.001 |
| APTT (seconds) | 1.0083 (1.0057, 1.011) | <0.001 |
| ALT (U/L) | 1.0002 (1.0001, 1.0003) | <0.001 |
| ALP (U/L) | 1.0017 (1.0014, 1.002) | <0.001 |
| AST (U/L) | 1.0001 (1.0001, 1.0002) | <0.001 |
| Total bilirubin (mg/dL) | 1.08 (1.06, 1.1) | <0.001 |
| LDH (U/L) | 1.0002 (1.0001, 1.0002) | <0.001 |
| Myocardial infarction |  |  |
| Yes vs. No | 1.06 (0.95, 1.2) | 0.302 |
| Dementia |  |  |
| Yes vs. No | 1.57 (1.3, 1.89) | <0.001 |
| Cerebrovascular disease |  |  |
| Yes vs. No | 1.27 (1.1, 1.48) | 0.001 |
| Chronic pulmonary disease |  |  |
| Yes vs. No | 0.96 (0.85,1.08) | 0.472 |
| Diabetes |  |  |
| Yes vs. No | 1.05 (0.93, 1.19) | 0.409 |
| Paraplegia |  |  |
| Yes vs. No | 1.33 (1.04, 1.7) | 0.025 |
| Renal disease |  |  |
| Yes vs. No | 1.34 (1.19, 1.5) | <0.001 |
| Metastatic solid tumor |  |  |
| Yes vs. No | 2.21 (1.78, 2.75) | <0.001 |
| LAR | 3.56 (3.08, 4.12) | <0.001 |

**Additional Table S5** A univariate Cox regression model evaluated the association between LAR and 365-day mortality in patients with hypertensive heart disease complicated by heart failure.

| **Item** | **HR (95% CI)** | **p-value** |
| --- | --- | --- |
| Gender |  |  |
| Male vs. Female | 0.92 (0.83, 1.02) | 0.109 |
| Age (years) | 1.03 (1.02, 1.03) | <0.001 |
| Race |  |  |
| White vs Non-White | 1.1 (0.99, 1.22) | 0.080 |
| Heart rate (beats/min) | 1.0026 (0.9994, 1.0058) | 0.111 |
| Systolic blood pressure (mmHg) | 0.98 (0.98, 0.98) | <0.001 |
| Diastolic blood pressure (mmHg) | 0.97 (0.97, 0.98) | <0.001 |
| Temperature (°C) | 0.85 (0.82, 0.88) | <0.001 |
| SpO_2_ (%) | 0.98 (0.97, 0.98) | <0.001 |
| Hematocrit (%) | 0.97 (0.97, 0.98) | <0.001 |
| Hemoglobin (g/dL) | 0.91 (0.89, 0.93) | <0.001 |
| Platelets (×10⁹/L) | 0.9986 (0.9981, 0.9992) | <0.001 |
| White blood cell count (×10⁹/L) | 1.0068 (1.0029, 1.0107) | <0.001 |
| Albumin (g/dL) | 0.55 (0.51, 0.6) | <0.001 |
| Anion gap (mmol/L) | 1.07 (1.05,1.08) | <0.001 |
| Bicarbonate (mmol/L) | 0.96 (0.96, 0.97) | <0.001 |
| Blood urea nitrogen (mg/dL) | 1.0087 (1.0071, 1.0103) | <0.001 |
| Calcium (mmol/L) | 0.93 (0.87, 0.99) | 0.017 |
| Chloride (mmol/L) | 0.99 (0.98, 1) | 0.005 |
| Creatinine (mg/dL) | 1.06 (1.04, 1.09) | <0.001 |
| Glucose (mg/dL) | 1.0011 (1.0001, 1.0021) | 0.028 |
| Sodium (mmol/L) | 0.9961 (0.9878, 1.0046) | 0.370 |
| Potassium (mmol/L) | 1.18 (1.09, 1.28) | <0.001 |
| INR | 1.21 (1.17, 1.26) | <0.001 |
| PT (seconds) | 1.02 (1.02, 1.02) | <0.001 |
| APTT (seconds) | 1.0073 (1.0049, 1.0097) | <0.001 |
| ALT (U/L) | 1.0002 (1.0001, 1.0003) | 0.004 |
| ALP (U/L) | 1.0017 (1.0014, 1.0019) | <0.001 |
| AST (U/L) | 1.0001 (1.0001, 1.0001) | <0.001 |
| Total bilirubin (mg/dL) | 1.08 (1.06, 1.1) | <0.001 |
| LDH (U/L) | 1.0001 (1.0001, 1.0002) | <0.001 |
| Myocardial infarction |  |  |
| Yes vs. No | 1.07 (0.97, 1.19) | 0.180 |
| Dementia |  |  |
| Yes vs. No | 1.64 (1.39, 1.93) | <0.001 |
| Cerebrovascular disease |  |  |
| Yes vs. No | 1.2 (1.05, 1.37) | 0.006 |
| Chronic pulmonary disease |  |  |
| Yes vs. No | 1.05 (0.95, 1.17) | 0.360 |
| Diabetes |  |  |
| Yes vs. No | 1.04 (0.93, 1.16) | 0.499 |
| Paraplegia |  |  |
| Yes vs. No | 1.32 (1.06, 1.65) | 0.012 |
| Renal disease |  |  |
| Yes vs. No | 1.43 (1.29,1.58) | <0.001 |
| Metastatic solid tumor |  |  |
| Yes vs. No | 2.35 (1.93, 2.86) | <0.001 |
| LAR | 2.91 (2.54, 3.33) | <0.001 |

**Additional Table S6: Supplementary Table S6** Subgroup analyses for the impact of LAR on hospital mortality in patients with hypertensive heart disease complicated by heart failure.

| **Subgroup** | **Variable** | **Total** | **Event (%)** | **HR (95% CI)** | **p for interaction** |
| --- | --- | --- | --- | --- | --- |
| Overall |  |  |  |  |  |
| Crude | LAR | 3019 | 726 (24) | 4.86 (4.12~5.73) | <0.001 |
| Adjust | LAR | 3019 | 726 (24) | 5.37 (4.53~6.37) | <0.001 |
| Age | LAR |  |  |  | 0.741 |
| <65 |  | 777 | 127 (16.3) | 5.34 (3.76~7.57) |  |
| ≥65 |  | 2242 | 599 (26.7) | 5.02 (4.16~6.05) |  |
| Gender | LAR |  |  |  | 0.029 |
| No |  | 1243 | 298 (24) | 6.03 (4.72~7.7) |  |
| Yes |  | 1776 | 428 (24.1) | 4.19 (3.37~5.21) |  |
| Race | LAR |  |  |  | 0.440 |
| White |  | 1154 | 294 (25.5) | 5.21 (4.15~6.54) |  |
| Non-white |  | 1865 | 432 (23.2) | 4.54 (3.57~5.77) |  |
| Myocardial infarct | LAR |  |  |  | 0.298 |
| No |  | 1876 | 440 (23.5) | 5.44 (4.36~6.79) |  |
| Yes |  | 1143 | 286 (25) | 4.37 (3.4~5.61) |  |
| Dementia | LAR |  |  |  | 0.125 |
| No |  | 2790 | 664 (23.8) | 5.03 (4.25~5.95) |  |
| Yes |  | 229 | 62 (27.1) | 2.82 (1.32~6.03) |  |
| Cerebrovascular disease | LAR |  |  |  | 0.188 |
| No |  | 2548 | 588 (23.1) | 5.13 (4.27~6.17) |  |
| Yes |  | 471 | 138 (29.3) | 3.95 (2.73~5.71) |  |
| Chronic pulmonary disease | LAR |  |  |  | 0.005 |
| No |  | 2043 | 508 (24.9) | 4.25 (3.5~5.16) |  |
| Yes |  | 976 | 218 (22.3) | 7.35 (5.37~10.07) |  |
| Diabetes | LAR |  |  |  | 0.078 |
| No |  | 2116 | 492 (23.3) | 5.52 (4.44~6.85) |  |
| Yes |  | 903 | 234 (25.9) | 4.15 (3.2~5.38) |  |
| Paraplegia | LAR |  |  |  | 0.231 |
| No |  | 2881 | 694 (24.1) | 4.95 (4.19~5.84) |  |
| Yes |  | 138 | 32 (23.2) | 2.94 (1.15~7.5) |  |
| Renal disease | LAR |  |  |  | 0.268 |
| No |  | 1369 | 284 (20.7) | 5.46 (4.16~7.16) |  |
| Yes |  | 1650 | 442 (26.8) | 4.53 (3.7~5.56) |  |
| Metastatic solid tumor | LAR |  |  |  | 0.703 |
| No |  | 2890 | 692 (23.9) | 4.84 (4.09~5.72) |  |
| Yes |  | 129 | 34 (26.4) | 5.41 (2.18~13.4) |  |

**Additional** **Table S7: Supplementary Table S6** Subgroup analyses for the impact of LAR on 30-day mortality in patients with hypertensive heart disease complicated by heart failure.

| **Subgroup** | **Variable** | **Total** | **Event (%)** | **HR (95% CI)** | **p for interaction** |
| --- | --- | --- | --- | --- | --- |
| Overall |  |  |  |  |  |
| Crude | LAR | 3019 | 894 (29.6) | 4.16 (3.56~4.87) | <0.001 |
| Adjust | LAR | 3019 | 894 (29.6) | 4.74 (4.02~5.58) | <0.001 |
| Age | LAR |  |  |  | 0.646 |
| <65 |  | 777 | 146 (18.8) | 4.66 (3.31~6.55) |  |
| ≥65 |  | 2242 | 748 (33.4) | 4.31 (3.61~5.15) |  |
| Gender | LAR |  |  |  | 0.028 |
| No |  | 1243 | 374 (30.1) | 5.14 (4.06~6.5) |  |
| Yes |  | 1776 | 520 (29.3) | 3.62 (2.94~4.45) |  |
| Race | LAR |  |  |  | 0.171 |
| White |  | 1154 | 339 (29.4) | 4.7 (3.76~5.86) |  |
| Non-white |  | 1865 | 555 (29.8) | 3.79 (3.03~4.75) |  |
| Myocardial infarct | LAR |  |  |  | 0.444 |
| No |  | 1876 | 546 (29.1) | 4.53 (3.66~5.61) |  |
| Yes |  | 1143 | 348 (30.4) | 3.85 (3.04~4.89) |  |
| Dementia | LAR |  |  |  | 0.076 |
| No |  | 2790 | 807 (28.9) | 4.34 (3.69~5.1) |  |
| Yes |  | 229 | 87 (38) | 2.42 (1.23~4.74) |  |
| Cerebrovascular disease | LAR |  |  |  | 0.536 |
| No |  | 2548 | 721 (28.3) | 4.27 (3.58~5.09) |  |
| Yes |  | 471 | 173 (36.7) | 3.81 (2.72~5.34) |  |
| Chronic pulmonary disease | LAR |  |  |  | 0.001 |
| No |  | 2043 | 612 (30) | 3.56 (2.94~4.3) |  |
| Yes |  | 976 | 282 (28.9) | 6.71 (5.02~8.97) |  |
| Diabetes | LAR |  |  |  | 0.343 |
| No |  | 2116 | 612 (28.9) | 4.44 (3.61~5.47) |  |
| Yes |  | 903 | 282 (31.2) | 3.85 (3.02~4.92) |  |
| Paraplegia | LAR |  |  |  | 0.546 |
| No |  | 2881 | 840 (29.2) | 4.21 (3.59~4.95) |  |
| Yes |  | 138 | 54 (39.1) | 3.53 (1.76~7.09) |  |
| Renal disease | LAR |  |  |  | 0.192 |
| No |  | 1369 | 349 (25.5) | 4.76 (3.68~6.17) |  |
| Yes |  | 1650 | 545 (33) | 3.86 (3.17~4.69) |  |
| Metastatic solid tumor | LAR |  |  |  | 0.762 |
| No |  | 2890 | 823 (28.5) | 4.09 (3.47~4.82) |  |
| Yes |  | 129 | 71 (55) | 5.57 (2.86~10.82) |  |

**Additional T****able S8: Supplementary Table S6** Subgroup analyses for the impact of LAR on 90-day mortality in patients with hypertensive heart disease complicated by heart failure.

| **Subgroup** | **Variable** | **Total** | **Event (%)** | **HR (95%CI)** | **p for interaction** |
| --- | --- | --- | --- | --- | --- |
| Overall |  |  |  |  |  |
| Crude | LAR | 3019 | 1178 (39) | 3.56 (3.08~4.12) | <0.001 |
| Adjust | LAR | 3019 | 1178 (39) | 4.07 (3.50~4.74) | <0.001 |
| Age | LAR |  |  |  | 0.843 |
| <65 |  | 777 | 192 (24.7) | 3.84 (2.79~5.28) |  |
| ≥65 |  | 2242 | 986 (44) | 3.75 (3.19~4.43) |  |
| Gender | LAR |  |  |  | 0.016 |
| No |  | 1243 | 487 (39.2) | 4.42 (3.54~5.52) |  |
| Yes |  | 1776 | 691 (38.9) | 3.1 (2.56~3.75) |  |
| Race | LAR |  |  |  | 0.208 |
| White |  | 1154 | 443 (38.4) | 3.95 (3.22~4.86) |  |
| Non-white |  | 1865 | 735 (39.4) | 3.3 (2.69~4.06) |  |
| Myocardial infarct | LAR |  |  |  | 0.119 |
| No |  | 1876 | 724 (38.6) | 4.09 (3.36~4.97) |  |
| Yes |  | 1143 | 454 (39.7) | 3.11 (2.49~3.9) |  |
| Dementia | LAR |  |  |  | 0.012 |
| No |  | 2790 | 1053 (37.7) | 3.77 (3.24~4.38) |  |
| Yes |  | 229 | 125 (54.6) | 1.83 (1.01~3.3) |  |
| Cerebrovascular disease | LAR |  |  |  | 0.666 |
| No |  | 2548 | 966 (37.9) | 3.63 (3.08~4.27) |  |
| Yes |  | 471 | 212 (45) | 3.34 (2.42~4.61) |  |
| Chronic pulmonary disease | LAR |  |  |  | 0.002 |
| No |  | 2043 | 805 (39.4) | 3.13 (2.63~3.72) |  |
| Yes |  | 976 | 373 (38.2) | 5.47 (4.13~7.24) |  |
| Diabetes | LAR |  |  |  | 0.370 |
| No |  | 2116 | 814 (38.5) | 3.77 (3.12~4.57) |  |
| Yes |  | 903 | 364 (40.3) | 3.32 (2.64~4.17) |  |
| Paraplegia | LAR |  |  |  | 0.683 |
| No |  | 2881 | 1112 (38.6) | 3.6 (3.1~4.18) |  |
| Yes |  | 138 | 66 (47.8) | 3.17 (1.64~6.13) |  |
| Renal disease | LAR |  |  |  | 0.199 |
| No |  | 1369 | 468 (34.2) | 4.04 (3.18~5.14) |  |
| Yes |  | 1650 | 710 (43) | 3.33 (2.78~3.99) |  |
| Metastatic solid tumor | LAR |  |  |  | 0.798 |
| No |  | 2890 | 1091 (37.8) | 3.51 (3.02~4.08) |  |
| Yes |  | 129 | 87 (67.4) | 4.64 (2.43~8.85) |  |

**Additional Table S9: Supplementary Table S6** Subgroup analyses for the impact of LAR on 365-day mortality in patients with hypertensive heart disease complicated by heart failure.

| **Subgroup** | **Variable** | **Total** | **Event (%)** | **HR (95%CI)** | **p for interaction** |
| --- | --- | --- | --- | --- | --- |
| Overall |  |  |  |  |  |
| Crude | LAR | 3019 | 1552 (51.4) | 2.91 (2.54~3.33) | <0.001 |
| Adjust | LAR | 3019 | 1552 (51.4) | 3.33 (2.89~3.83) | <0.001 |
| Age | LAR |  |  |  | 0.391 |
| <65 |  | 777 | 283 (36.4) | 2.75 (2.05~3.68) |  |
| ≥65 |  | 2242 | 1269 (56.6) | 3.17 (2.72~3.71) |  |
| Gender | LAR |  |  |  | 0.017 |
| No |  | 1243 | 663 (53.3) | 3.59 (2.92~4.41) |  |
| Yes |  | 1776 | 889 (50.1) | 2.55 (2.13~3.05) |  |
| Race | LAR |  |  |  | 0.070 |
| White |  | 1154 | 564 (48.9) | 3.35 (2.76~4.07) |  |
| Non-white |  | 1865 | 988 (53) | 2.64 (2.18~3.2) |  |
| Myocardial infarct | LAR |  |  |  | 0.028 |
| No |  | 1876 | 950 (50.6) | 3.43 (2.85~4.12) |  |
| Yes |  | 1143 | 602 (52.7) | 2.44 (1.98~3.01) |  |
| Dementia | LAR |  |  |  | 0.029 |
| No |  | 2790 | 1391 (49.9) | 3.04 (2.64~3.5) |  |
| Yes |  | 229 | 161 (70.3) | 1.75 (1.05~2.91) |  |
| Cerebrovascular disease | LAR |  |  |  | 0.835 |
| No |  | 2548 | 1288 (50.5) | 2.9 (2.49~3.38) |  |
| Yes |  | 471 | 264 (56.1) | 2.98 (2.21~4.02) |  |
| Chronic pulmonary disease | LAR |  |  |  | 0.035 |
| No |  | 2043 | 1030 (50.4) | 2.71 (2.31~3.18) |  |
| Yes |  | 976 | 522 (53.5) | 3.93 (3.01~5.13) |  |
| Diabetes | LAR |  |  |  | 1.000 |
| No |  | 2116 | 1079 (51) | 2.91 (2.43~3.48) |  |
| Yes |  | 903 | 473 (52.4) | 2.92 (2.36~3.61) |  |
| Paraplegia | LAR |  |  |  | 0.376 |
| No |  | 2881 | 1468 (51) | 2.88 (2.5~3.31) |  |
| Yes |  | 138 | 84 (60.9) | 3.77 (2.12~6.68) |  |
| Renal disease | LAR |  |  |  | 0.258 |
| No |  | 1369 | 605 (44.2) | 3.23 (2.57~4.05) |  |
| Yes |  | 1650 | 947 (57.4) | 2.78 (2.35~3.29) |  |
| Metastatic solid tumor | LAR |  |  |  | 0.655 |
| No |  | 2890 | 1445 (50) | 2.86 (2.48~3.29) |  |
| Yes |  | 129 | 107 (82.9) | 4.08 (2.19~7.61) |  |
